# Polygalacturonase gene *pgxB* in *Aspergillus niger* is a virulence factor in apple fruit

**DOI:** 10.1371/journal.pone.0173277

**Published:** 2017-03-03

**Authors:** Cheng-Qian Liu, Kang-Di Hu, Ting-Ting Li, Ying Yang, Feng Yang, Yan-Hong Li, He-Ping Liu, Xiao-Yan Chen, Hua Zhang

**Affiliations:** 1 School of Food Science and Engineering, Hefei University of Technology, Hefei, China; 2 College of Environment and Energy Engineering, Anhui Jianzhu University, Hefei, China; 3 Xuzhou Institute of Agricultural Sciences of the Xuhuai District of Jiangsu Province, Xuzhou, China; 4 Anhui Siping Food Development Co. Ltd., Tongling, China; Universita degli Studi di Pisa, ITALY

## Abstract

*Aspergillus niger*, a saprophytic fungus, is widely distributed in soil, air and cereals, and can cause postharvest diseases in fruit. Polygalacturonase (PG) is one of the main enzymes in fungal pathogens to degrade plant cell wall. To evaluate whether the deletion of an exo-polygalacturonase gene *pgxB* would influence fungal pathogenicity to fruit, *pgxB* gene was deleted in *Aspergillus niger* MA 70.15 (wild type) via homologous recombination. The Δ*pgxB* mutant showed similar growth behavior compared with the wild type. Pectin medium induced significant higher expression of all pectinase genes in both wild type and Δ*pgxB* in comparison to potato dextrose agar medium. However, the Δ*pgxB* mutant was less virulent on apple fruits as the necrosis diameter caused by Δ*pgxB* mutant was significantly smaller than that of wild type. Results of quantitive-PCR showed that, in the process of infection in apple fruit, gene expressions of polygalacturonase genes *pgaI*, *pgaII*, *pgaA*, *pgaC*, *pgaD* and *pgaE* were enhanced in Δ*pgxB* mutant in comparison to wild type. These results prove that, despite the increased gene expression of other polygalacturonase genes in Δ*pgxB* mutant, the lack of *pgxB* gene significantly reduced the virulence of *A*. *niger* on apple fruit, suggesting that *pgxB* plays an important role in the infection process on the apple fruit.

## Introduction

Pectinases are the most important pathogenic factor in plant pathogenic bacteria and fungi [1–4]. They are responsible for pathogens to decompose pectin in plant cell wall. Pectin hydrolysis not only weakens the cell wall to facilitate penetration and colonization of the host, it also provides the fungus carbon sources for its growth [5]. Pectinases are consisted of polygalacturonase (PG), pectin lyase (PNL), pectate lyase (PL), pectinesterase (PE), pectin methyl esterase (PME). Polygalacturonase is one of the major members of pectinases which cleaves α-1,4-glycosidic of D-galacturonic acid in pectin and it is classified into endo- and exo-polygalacturonase on the basis of the way of eliminating galacturonic acid [6,7].

The production of PG by pathogenic fungi is critical for their success and survival during host infection [8]. It has been confirmed that the loss of a polygalacturonase gene in some fungi would result in decreased pathogenicity. Shieh et al [9] showed that a polygalacturonase gene is related to the infection of *Aspergillus flavus* in cotton bolls. The disruption of endo-polygalacturonase gene *Bcpg1* or *Bcpg2* in *Botrytis cinerea* reduced its virulence on different hosts [10]. It is also reported that the loss of pectin methyl esterase gene *Bcpme1* reduced virulence on apples, and pectin lyase *pel*B was an important virulence factor in *Colletotrichum gloeosporioides* when attacking avocado [11,12]. PG is also required for infection by *Phytophthora capsici* and *Alternaria citri* [13,14]. However there are also studies demonstrated that disruption of some polygalacturonase genes in fungi did not directly affect virulence, for example, deletion of *PG1*, *PG5*, *PGX4* in *Fusarium oxysporum* led to no virulence difference in tomato [15,16] and endopolygalacturonase *PGN1* is not required for pathogenicity of *Cochliobolus carbonum* on maize [17]. Mutants lacking both polygalacturonase genes *cppg1/cppg2* in *Claviceps purpurea* did not affected vegetative properties, but they are nearly nonpathogenic on rye [18].

*Aspergillus niger* is a saprophytic fungus which degrades plant cell wall polysaccharides and leads to the decay of fruits and vegetables [19]. Since the 1990s, technical advances in molecular biology speed up the operation mechanism of *A*. *niger* and *A*. *niger* gene sequencing has been completed [20,21]. Deletion of *kusA* gene in *A*. *niger* dramatically improved homologous integration efficiency and facilitated gene knockout in *A*. *niger* [22]. However, whether polygalacturonase contributes to the pathogenicity of *A*. *niger* on fruit is still unclear. Here we constructed *pgxB* deletion mutant in *A*. *niger* MA 70.15 via homologous recombination and the pathogenicity were evaluated in Δ*pgxB* strain.

## Material and methods

### Fungal strains and growth conditions

*A*. *niger* MA 70.15 (Δ*pyrG*, *pyrG* encodes orotidine-5-phosphate decarboxylase, cell lacking this enzyme cannot grow without exogenous uridine, but can resist toxicity of 5-Fluoroorotic acid) was used as wild type strain in this work. *A*. *niger* was grown on potato dextrose agar medium (PDA) (per liter: 200 g of potato; 20 g of agar; 20 g of glucose and 10 mM uridine) at 28°C. Therefore all medium used in this study were supplemented with 10 mM uridine except where stated. For in vitro growth evaluation, *A*. *niger* spores were suspended in sterile water and adjusted to 10^6^ spores per mL. To test whether the absence of *pgxB* affected mycelial growth, PDA and pectolytic enzyme-inducing medium (PEIM) (per liter: 20 g of agar; 20 g of pectin; MM medium (5 g of KNO_3_; 0.3 g of KCl; 2 g of MgSO_4_·7H_2_O; 5 g of KH_2_PO_4_; 0.008 mg of Na_2_B_4_O_7_·10H_2_O; 0.16 mg CuSO_4_·5H_2_O; 0.256 mg of FeCl_3_·6H_2_O; 0.1213 mg of MnSO_4_·4H_2_O; 0.16 mg of NaMoO_4_·2H_2_O; 2.85 mg of ZnSO_4_) plus 10 mM uridine) [23] were spot-inoculated with 5 μL spore suspension of wild type and Δ*pgxB* mutant. All strains were inoculated onto three plates of each medium and colony diameter was measured daily.

For the determination of growth curve, 1 mL spore suspension of wild type and Δ*pgxB* mutant were inoculated in 100 mL Erlenmeyer flasks containing 30 mL potato dextrose medium or PEIM at 30°C, 150 rpm. Mycelium was harvested on a quantitative filter paper at the time of 12, 24, 36, 48, 60, 72, 84, 96 h and weighed.

### Vector construction and transformation

The exo-polygalacturonase gene *pgxB* in *A*. *niger* was deleted following gene knockout method of Delmas et al [24]. Upstream and downstream DNA fragments AB (548 bp) and CD (564 bp) flanking *pgxB* gene were amplified by polymerase chain reaction (PCR) from *A*. *niger* MA 70.15 genomic DNA. Primers were designed using the genome database *A*. *niger* CBS 513.88 and the upstream and downstream fragments contained a common *Hind*III restriction site to ligate them together and *Eco*RI and *Xho*I restriction sites were used for cloning the joined fragment into the plasmid pC3 [24] to create pC3-*An_*Δ*pgxB* integrative plasmid. Primers are shown in Table 1.

**Table 1:** Primers used for gene knockout and quantitative PCR.

Primer	sequence
used for gene knockout and PCR confirmation	
*pgxB*-A (forward)	5'-TTGCGGCCGCTTTTGCGTCTTGATTGTGAG-3'
*pgxB*-B (reverse)	5'-CGACAGACCCAAGCTTTGATGTGGGTAGATGCGTAG-3'
*pgxB*-C (forward)	5'-TTAAGCTTGGGTCTGTCGTTGATGATTT-3'
*pgxB*-D (reverse)	5'-TTACTAGTTGTTCGAGAAGGGTGGTTTT-3'
*pgxB*-E1 (forward)	5'-GCTTTCGGCGTCTCAATC-3'
*pgxB*-E2 (reverse)	5'-TTCGGCGAGAAGCAGTCAT-3'
used for quantitative PCR	First line: forward/ second line: reverse
*pgxB*	5'-TTCGGCGAGAAGCAGTCA-3'
	5'-CACCGATAATGCAACCCG-3'
*pgaI*	5'-CATGAACTTGGGCTTGGTC-3'
	5'-TGATCCGCTTCGGTGGTA-3'
*pgaII*	5'-TGGCTCCGTGTCCGAGA-3'
	5'-ATCGTCCCAGGTCCAGTCC-3'
*pgaA*	5'-CGTTGAGGTCCTTCAGGTTAA-3'
	5'-AGCCTTTGTTCTGCCTTGC-3'
*pgaB*	5'-GGACCCTTCCATTCCTTGTA-3'
	5'-TTTCACCTCCGCCTCTGC-3'
*pgaC*	5'-GCCCTTCCCATTCCTCG-3'
	5'-TCCCATCTGGCACAACCC-3'
*pgaD*	5'-TAAGGGCATCCCGAAGAG-3'
	5'-ATGTCAAGGGTACTATGAGCG-3'
*pgaE*	5'-ACTTGGGCTTCGTCTTGC-3'
	5'-TGGAGCGGACCTCTTGTC-3'
*pgaX*	5'-ACCTCGTCCTTGTATTGGC-3'
	5'-GGTGATGACTGCGTCTCCT-3'
*pgxA*	5'-GCGACGACAAAGTCACCC-3'
	5'-GCCAGAACAGCACAAGCA-3'
*pgxC*	5'-GGCTGCCGTTGCGTTCAT-3'
	5'-TGCGTTTCCGACCCTTGC-3'
*pelA*	5'-GCTGACAATACGGAGACCCT-3'
	5'-GGACGACTGGTGCGAGAA-3'
*pelB*	5'-TGCCCTGACCAACAATACTC-3'
	5'-GAACTGCTTCCCAATGCC-3'
*pelC*	5'-AGGGAGGTCTCGCAGTGGTC-3'
	5'-GCTCAGCGGAAAGGGTCT-3'
*pelD*	5'-CCCGAGTTGTTCTCCCAGT-3'
	5'-ACGTGTACCTTGACGGCTCT-3'
*pelF*	5'-GCACCAGTCGGAGCCATT-3'
	5'-CCCCGTGTCATCCTTATCG-3'
*plyA*	5'-GGCGGTCTGCTTGATGGTA-3'
	5'-GCTGCGTTCGGCTATGC-3'
*pmeA*	5'-GCTGGTGTAGGTGGTGGTGT-3'
	5'-TTTTCTTTGCGGCGACTG-3'
*An04g09690*	5'-TCGCTTCTTGATGTGCTCTGC-3'
	5'-GTTCACTTGGCGTGCCTGTC-3'
*An02g12505*	5'-CCTCCTCGCTGCTACTCAA-3'
	5'-TCGGGATACATCAACTGGG-3'
*Actin*	5'-ACGCTTGGACTGTGCCTC-3'
	5'-CAATGGTTCGGGTATGTGC-3'

Underlined letters refer to restriction enzymes site.

*A*. *niger* protoplast preparation and transformation were carried out by the method of Baltz et al [23]. 4-day-old mycelia grown from PDA were harvested and were digested with 0.4 g Lyticase (Sigma) to obtain fungal protoplasts. Protoplasts were transformed with 10 μg pC3-*An*_Δ*pgxB* (un-linearized) in 50 μL polyethylene glycol 6000. Transformations were inoculated on upper layer of transformation medium without uridine (per liter: upper medium: MM; 6 g of agar; 0.95 M sucrose; lower medium: MM; 12 g of agar; 0.95 M sucrose) to select for the integration of the plasmid carrying *pyrG* on the chromosome. Transformants were purified by breeding them twice successively on the same transformation medium but lacking sorbitol (per liter: MM, 20 g of agar). Transformants were then propagated twice on PDA medium containing 10 mM uridine to release the selective pressure on the integrated plasmid. For selecting clones that had excised the plasmid (Δ*pyrG*), spores were then spread on MM-Uri-5-FOA medium (per liter: MM; 20 g of agar; 10 g of glucose; 1.6 mM uridine; 750 μg/mL 5-fluoro-orotic acid). Clones from last medium were cultured in MM-Uri-5-FOA liquid medium at 250 rpm at 30°C for 3 d, and mycelia were harvested for genomic DNA extraction.

### DNA extraction and PCR confirmation of Δ*pgxB* strain

Genomic DNA was extracted using Master Pure Yeast DNA Purification Kit (Epicentre). Primers *pgxB*-A, *pgxB*-D and another primers internal to the *pgxB* gene *pgxB*-E1, *pgxB*-E2 (PCR product size: 953 bp) were designed using Primer Premier 5.0 software. Primers are shown in Table 1.

### Determination of PG activity

For determination of polygalacturonase activity, 100 μL of conidia (1×10^6^ spores per mL) were inoculated into a 100 mL Erlenmeyer flask containing 30 mL of liquid PEIM and cultured at 30°C for 5 days. Culture medium after suction filtration was used for PG activity assay following the method described by Miller [25]. Reaction mixture was consisted of 1 mL of 50 mM acetic acid-sodium acetate at pH5.5, 0.5 mL of 10 g/L pectin solution and 0.5 mL of crude enzyme or enzyme boiled for 5 min followed by incubation at 40°C for 30 min. Reactions were terminated by adding 1.5 mL of DNS (3,5-dinitrosalicylic acid) followed by a 5 min incubation in a boiling water bath. The reaction mixture was cooled to room temperature, and distilled water was added to a final volume of 25 mL. Absorbance at 540 nm was measured. One unit of PG was defined as 1 μg of galacturonic acid produced by pectin per hour and expressed as U/mL enzyme extract.

PG activity was also determined by plate assay. The wild type and Δ*pgxB* mutant were inoculated on PEIM and cultured for 3 days at 30°C. Thereafter the colonies were rinsed off the plates with distilled water before staining the plates with 0.05% ruthenium red. Pectinase production was evaluated by ratio of diameter of clear zone formed around colonies relative to diameter of mycelia [26,27].

### Virulence assay of *A*. *niger* on apple fruit

Apple fruits were washed with tap water and then surface-sterilized with 75% ethanol. Five different sites on the surface of apples were wounded (2 mm diameter and 5 mm deep) and injected with 5 μL wild type or Δ*pgxB* mutant spore suspension (10^6^ spores per mL) respectively. After air-drying, three replicates were put in sealed container with H_2_O at the bottom at 25°C. The necrosis diameter were measured daily after inoculation. Spores grown from wounds were used for RNA extraction and quantitative PCR.

### RNA extraction and quantitative PCR

RNA was extracted from frozen mycelium grown on PDA, PEIM or apples ground in liquid nitrogen with TRNzol RNA Reagent kit (Tiangen). After DNase treatment, cDNA were obtained according to the manufacturer’s instruction of PrimeScript RT Master Mix (TaKaRa, Japan). Primers used for quantitative PCR are shown in Table 1. Relative quantification was processed using the method of Delta-Ct.

### Statistical analysis

Statistical significance was tested by one-way analysis of variance (ANOVA), and the results are expressed as the mean values ± standard deviation (SD) of three independent experiments. Fisher’s least significant differences (LSD) were calculated following a significant (*P* < 0.01 or *P* < 0.05) *t* test.

## Results

### Gene disruption of *pgxB* in *A*. *niger*

548-bp upstream and 564-bp downstream DNA fragments AB and CD were amplified by PCR from *A*. *niger* MA 70.15 genomic DNA. Fragment ABCD (1094 bp) by the ligation of AB and CD were cloned into plasmid pC3 to generate the recombinant plasmid pC3-*An_*Δ*pgxB* (6959 bp).

**Fig 1:**
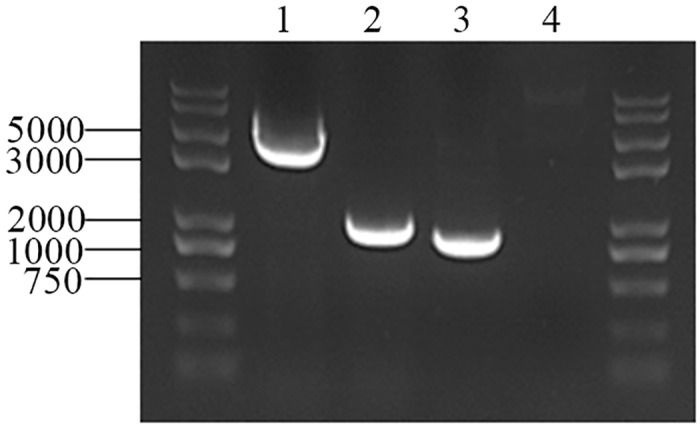
Confirmation of *pgxB* deletion mutant by PCR analysis of genomic DNA from wild type and Δ*pgxB*. Lane 1 and 2, genomic PCR of *A*. *niger* MA 70.15 and Δ*pgxB* with external primers respectively; lane 3 and 4, genomic PCR of *A*. *niger* MA 70.15 and Δ*pgxB* with internal primers respectively.

Then the plasmid pC3-*An_*Δ*pgxB* was transformed and integrated into *A*. *niger* MA 70.15 protoplast and the Δ*pgxB* mutant was confirmed by genomic PCR. As shown in Fig 1, genomic DNA was used as template for PCR confirmation with primers *pgxB*-A and *pgxB*-D which flank *pgxB* gene and primers on the ORF of *pgxB* gene *pgxB*-E1 and *pgxB*-E2. 1100 bp band for Δ*pgxB* and 3500 bp for the wild type were obtained by the primers *pgxB*-A and *pgxB*-D, and a 953-bp band for the wild type and no amplification for Δ*pgxB* with the primers *pgxB*-E1 and *pgxB*-E2. All of these results confirmed that the *pgxB* gene was disrupted in Δ*pgxB*.

### Growth analysis of Δ*pgxB* mutant strain

**Fig 2:**
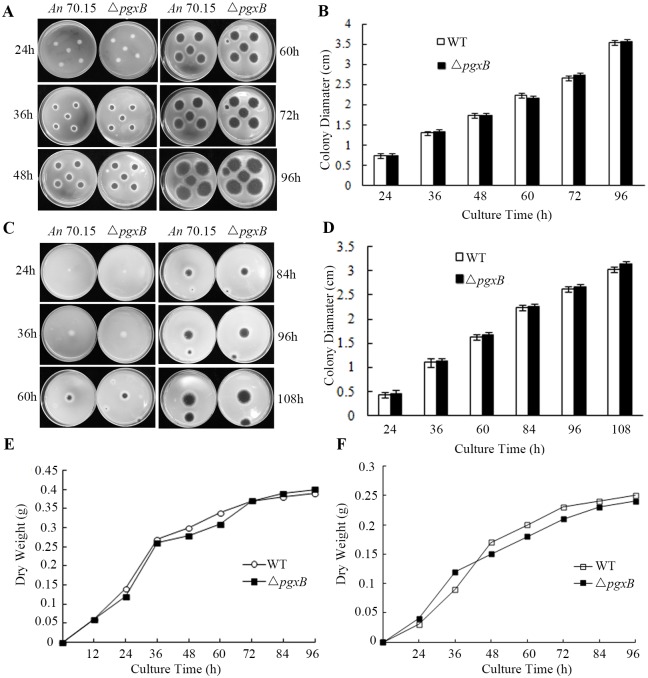
Radial mycelia growth of Δ*pgxB* strain on PDA and PEIM medium. A and B show mycelia growth and diameter analysis of the wild type *A*. *niger* MA 70.15 (WT) and Δ*pgxB* mutant on PDA for 96 h; C and D show mycelia growth and mycelia diameter of the wild type *A*. *niger* MA 70.15 (WT) and Δ*pgxB* mutant on PEIM for 108 h. E and F, dry weight of the wild type *A*. *niger* MA 70.15 (WT) and Δ*pgxB* strain in potato dextrose and pectolytic enzyme-inducing liquid medium for 96 h.

Pectinases in some fungi can be induced by pectin [28]. To evaluate whether the lack of *pgxB* would affect its growth on pectin medium, we compared growth of Δ*pgxB* and *A*. *niger* MA 70.15 on PDA (no pectin) and pectin medium (PEIM). The radial growth was measured on solid media. It was found that growth rate, estimated as colony diameter, showed no difference between the Δ*pgxB* mutant and wild type on PDA (Fig 2A and 2B) or pectin medium (Fig 2C and 2D). Besides, no significant difference in growth curve was found between Δ*pgxB* mutant and the wild type in liquid medium with or without pectin (Fig 2E and 2F).

### Inducing effect of pectin on the expression of pectinase genes in *A*. *niger*

**Fig 3:**
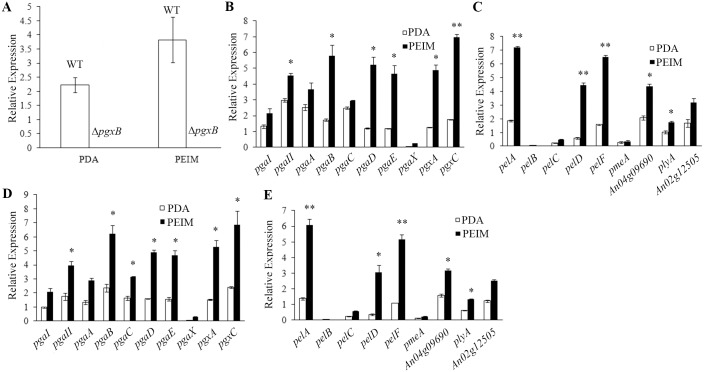
Pectin medium (PEIM) induces higher expression of pectinase genes in wild type and Δ*pgxB* mutant grown on PDA and PEIM. A, relative expression of *pgxB* in *A*. *niger* MA 70.15 and Δ*pgxB* mutant on PDA and PEIM; B and C, relative expression of *pgaI*, *pgaII*, *pgaA*, *pgaB*, *pgaC*, *pgaD*, *pgaE*, *pgaX*, *pgxA*, *pgxC*, *pelA*, *pelB*, *pelC*, *pelD*, *pelF*, *pmeA*, *An04g09690*, *plyA* and *An02g12505* in *A*. *niger* MA 70.15 on PDA and PEIM medium respectively for 4 days; C and D, relative expression of *pgaI*, *pgaII*, *pgaA*, *pgaB*, *pgaC*, *pgaD*, *pgaE*, *pgaX*, *pgxA*, *pgxC*, *pelA*, *pelB*, *pelC*, *pelD*, *pelF*, *pmeA*, *An04g09690*, *plyA* and *An02g12505* in Δ*pgxB* mutant on PDA and PEIM medium respectively for 4 day**s**. * and ** in this figure and following ones stand for a significant difference between two data at *P* < 0.05 and *P* < 0.01, respectively.

To study whether the expression of pectinase genes were induced by pectin, relative expression of different pectinase genes in Δ*pgxB* and *A*. *niger* MA 70.15 on PDA and pectin medium (PEIM) were determined by quantitative PCR. As shown in Fig 3A, expression of *pgxB* gene in wild type was significantly enhanced by pectin medium, while the expression was not detected in Δ*pgxB*, further confirming that *pgxB* gene was deleted in the mutant. For wild type, most of pectinase genes like PG genes *pgaII*, *pgaB*, *pgaD*, *pgaE*, *pgaX*, *pgxA*, *pgxC*, PL genes *pelA*, *pelC*, *pelD*, *pelF*, *plyA*, PME gene *An04g09690*, PE gene *An02g12505* showed enhanced expression on PEIM than those on PDA, suggesting that the expression of pectinases genes were induced by pectin (Fig 3B and 3C). Similarly, enhanced gene expression of PG genes *pgaII*, *pgaA*, *pgaB*, *pgaD*, *pgaE*, *pgaX*, *pgxA*, *pgxC*, PL genes *pelA*, *pelC*, *pelD*, *pelF*, *plyA*, PME gene *An04g09690*, PE gene *An02g12505* was also observed in Δ*pgxB* mutant (Fig 3D and 3E).

### Polygalacturonase activity analysis

**Fig 4:**
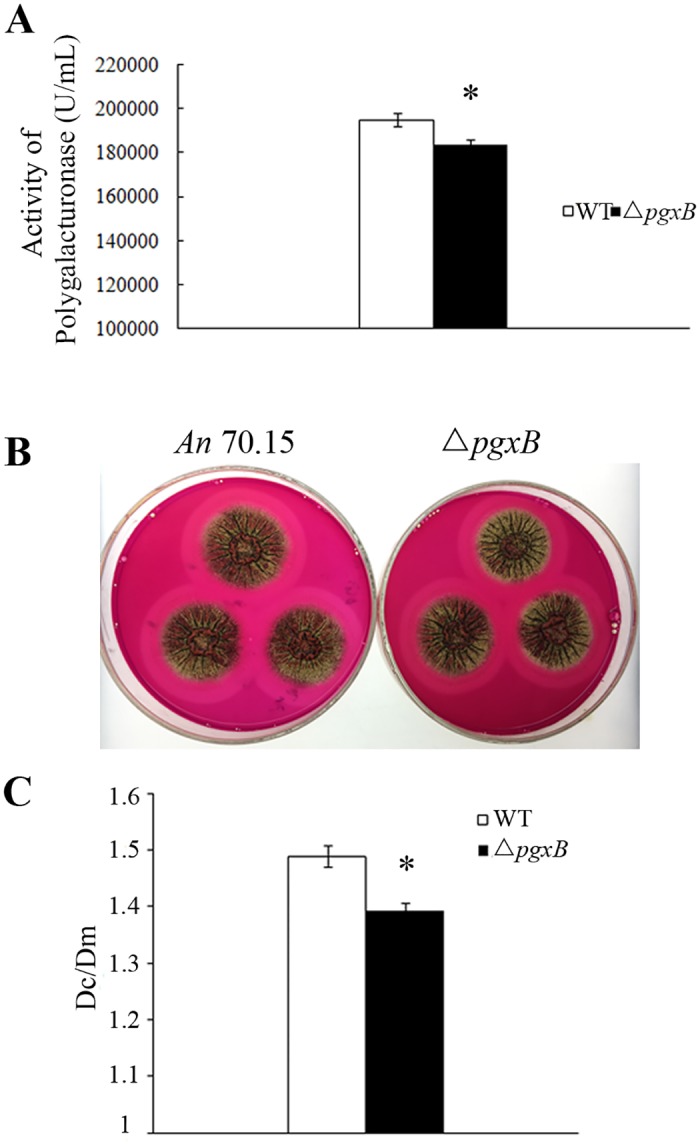
Activity evaluation of secreted polygalacturonase (PG) from *A*. *niger* MA 70.15 and Δ*pgxB* mutant. A, the wild type *A*. *niger* MA 70.15 (WT) and Δ*pgxB* strains were grown in liquid pectolytic enzyme-inducing media (PEIM) for 5 days and 0.5 mL supernatant of PEIM cultures was sampled for polygalacturonase activity assay. B, The wild type and Δ*pgxB* mutant were inoculated on pectolytic enzyme-inducing media (PEIM) and cultured for 3 days at 30°C and the plates were stained with 0.05% ruthenium red and photographed. C, ratio of diameter of activity zone (clear zone, Dc) relative to the diameter of mycelia (Dm).

*pgxB* gene is a member of polygalacturonase family found in *A*. *niger*. To study whether *pgxB* gene actually contributes to the whole activity of polygalacturonase in *A*. *niger*, polygalacturonase activity was determined in Δ*pgxB* mutant. The Δ*pgxB* mutant and *A*. *niger* MA 70.15 were grown in liquid PEIM for 3 days and the activity of secreted polygalacturonase in the medium was analyzed. As shown in Fig 4A, Δ*pgxB* exhibited lower PG activity (by 5.8%) compared with the wild type. Secreted PG activity by *A*. *niger* was also assayed on plate. As shown in Fig 4B and 4C, the ratio of diameter of clear zone (Dc) which showing the degradation of pectin by secreted PG and mycelia (Dm) produced by Δ*pgxB* was smaller (by 6.5%) than that of wild type, indicating that Δ*pgxB* mutant produced less pectinase than the wild type *A*. *niger* MA 70.15.

### Pathogenicity assay on apple fruit

**Fig 5:**
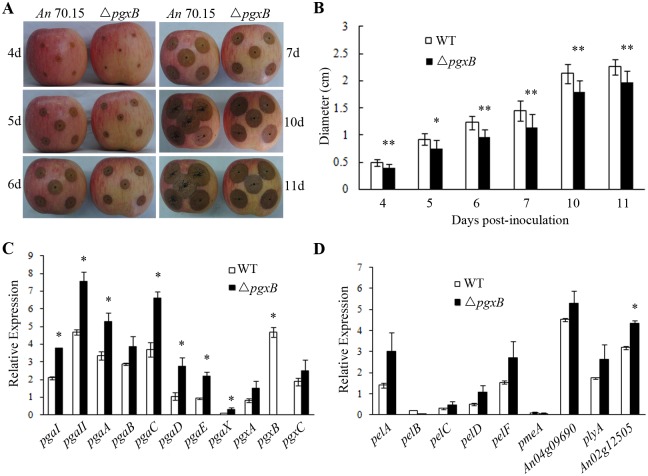
Pathogenicity assay on apples with the wild type and Δ*pgxB* mutant. A and B, the wild type *A*. *niger* MA 70.15 and Δ*pgxB* mutant grown on apples at 4, 5, 6, 7, 10 and 11 days post-inoculation and the lesion diameter caused by wild type and Δ*pgxB* are shown. C and D, relative expression of different pectinase genes in *A*. *niger* MA 70.15 (WT) and Δ*pgxB* mutant after infecting apples for 16 days.

Whether pectinase was a pathogenic factor in *A*. *niger* infection on fruit is still unknown, thus we studied the virulence of Δ*pgxB* mutant on fruit. Apple fruits were inoculated with conidial suspension of Δ*pgxB* and the wild type *A*. *niger* MA 70.15. Lesion development was monitored daily and the diameter was measured. As shown in Fig 5A and 5B, *pgxB* disruption resulted in a reduction of decay development as the lesion diameter caused by Δ*pgxB* was about 20% smaller than that of wild type on 4, 5, 6, 7 days and 15% smaller on 10, 11 days, suggesting that the virulence of Δ*pgxB* on apple fruit was significantly lower than that of wild type.

To study why the virulence was decreased in Δ*pgxB*, expression of various pectinase genes in Δ*pgxB* and wild type in the process of infecting apples was determined by quantitative PCR. Expression of PG genes *pgaI*, *pgaII*, *pgaA*, *pgaC*, *pgaD*, *pgaE*, *pgaX* in Δ*pgxB* were higher in Δ*pgxB* than those in wild type, while there was no significant difference in expression of PG genes *pgaB*, *pgxA*, *pgxC*, PL genes *pelA*, *pelB*, *pelC*, *pelD*, *pelF*, *plyA* and PME genes *pmeA*, *An04g09690* between Δ*pgxB* mutant and wild type (Fig 5C and 5D). The increased expression of PG genes in Δ*pgxB* suggested a possible compensation effect in *pgxB* deletion mutant.

## Discussion

Four exo-polygalacturonase genes were found in *A*. *niger*, including *pgxA*, *pgxB*, *pgxC* and *pgaX* [29]. Besides, endo-polygalacturonase gene *pgaI*, *pgaII*, *pgaA*, *pgaB*, *pgaC*, *pgaD*, *pgaE*, pectin lyase gene *pelA*, *pelB*, *pelC*, *pelF*, *plyA*, pectin methylesterase gene *pmeA*, *An04g09690* have been isolated and sequenced from *A*. *niger* [30–36]. The gene *pgxB* in *A*. *niger* (gene ID: 4980661), consisting of 439 amino acids, with 5 exons and 4 introns, a molecular mass of 67 kDa, encodes the extracellular exo-polygalacturonase and it was studied in the present research.

In this paper, we described the construction of a mutant disrupted in the *pgxB* gene in *A*. *niger*. Δ*pgxB* exhibited no growth rate reduction on PDA and pectin medium compared with wild type, which means the disruption of *pgxB* did not weaken its ability of decomposing pectin as carbon source (Fig 2). In contrast, *Bcpme1* mutant in *B*. *cinerea* and *pel*B mutant in *Colletotrichum gloeosporioides* showed reduced growth on pectin medium [11,12]. Pectinase was induced by pectin, polygalacturonic acid or galacturonic acid and was repressed by glucose and polygalacturonase-inhibiting protein (PGIP) [37,38]. Quantitative PCR results showed that expression of most of pectinase genes such as PG genes *pgaII*, *pgaA*, *pgaB*, *pgaD*, *pgaE*, *pgaX*, *pgxA*, *pgxC*, PL genes *pelA*, *pelC*, *pelD*, *pelF*, *plyA* were up-regulated on pectin medium compared that on PDA (Fig 3), confirming that the expression of pectinase genes were induced by pectin. We also found that Δ*pgxB* mutant grown in liquid and solid PEIM showed significant lower PG activity than the wild-type strain, suggesting that the loss of *pgxB* reduced the production of PG (Fig 4).

Virulence assay on apple fruit showed that the deletion of *pgxB* gene has a profound effect on lesion development in the infection of apple as lesion diameter caused by Δ*pgxB* was smaller than that of wild type. A similar reduction in maceration ability has been observed with pectinase-deficient mutants of phytopathogenic bacteria such as *Erwinia*, *Pseudomonas* and *Ralstonia* species [39,40]. Besides, Oeser et al. [18] found that mutant in both *cppg1* and *cppg2* are nearly non-pathogenic on rye using a gene-replacement approach. However, pectinases are usually encoded by multiple genes, thus mutation in one pectinase gene might not affect the virulence on fruits [15–17]. The disruption of either the *pelA* or *pelD* gene in *Nectria hematococca* alone causes no detectable decrease in virulence, whereas disruption of both *pelA* and *pelD* drastically reduces virulence [41]. In order to understand the decreasing virulence of Δ*pgxB* mutant, expression of some pectinase genes were assayed, and we found that PG genes *pgaI*, *pgaII*, *pgaA*, *pgaC*, *pgaD*, *pgaE* and *pgaX* were expressed moore highly in Δ*pgxB* mutant than in the wild type. Deletion of one gene in a gene family might result in higher expression of other genes with the same function as polygalacturonases of *A*. *niger* are encoded by a family of diverged genes [42]. A similar phenomenon that disruption of serine proteinase caused an increase in metalloproteinase has also been found in *Aspergillus flavus* [43]. Even so, the lack of *pgxB* still dramatically reduced lesion diameter on apples. Our results demonstrate that *pgxB* is a virulence factor which partially contributes to the virulence of *A*. *niger* on apple fruit, thus highlighting the need for further research to elucidate the roles of other pectinase genes in *A*. *niger*.
