# Correction: Polygalacturonase gene *pgxB* in *Aspergillus niger* is a virulence factor in apple fruit

**DOI:** 10.1371/journal.pone.0191350

**Published:** 2018-01-11

**Authors:** Cheng-Qian Liu, Kang-Di Hu, Ting-Ting Li, Ying Yang, Feng Yang, Yan-Hong Li, He-Ping Liu, Xiao-Yan Chen, Hua Zhang

The Funding section contains incomplete information. The complete funding information is as follows: This work was supported by National Natural Science Foundation of China (31470013, 31670278 to HZ; 31300133 to KH), Anhui Provincial Science and Technology Major Project (6030701073 to HZ) and Anhui Provincial Education Department (KJ2015ZD12 to YY). The funders had no role in study design, data collection and analysis, decision to publish, or preparation of the manuscript. Anhui Siping Food Development Co. Ltd. provided support in the form of salaries for authors YHL and HPL, but did not have any additional role in the study design, data collection and analysis, decision to publish, or preparation of the manuscript. The specific roles of these authors are articulated in the 'author contributions' section.
